# Retrospective assessment of the predictors of neonatal and infantile cholestasis with and without liver failure: an experience from Southeast China

**DOI:** 10.7717/peerj.20800

**Published:** 2026-02-10

**Authors:** Yijun Lin, Rui Zhang, Weijie Ou, Hong Ye

**Affiliations:** 1 Department of Pediatrics, Fujian Maternity and Child Health Hospital, College of Clinical Medicine for Obstetrics & Gynecology and Pediatrics, Fujian Medical University, Fuzhou, China; 2 Department of Pediatrics, Fujian Children’s Hospital (Fujian Branch of Shanghai Children’s Medical Center), Fuzhou, China

**Keywords:** Neonatal, Infantile, Cholestasis, Liver failure, Predictor

## Abstract

**Background:**

Neonatal and infantile cholestasis with liver failure (LF) is a life-threatening condition. To identify predictive factors, it is essential to develop and validate novel nomograms for predicting neonatal and infantile cholestasis with LF separately in Southeast China.

**Methods:**

The medical records of neonates and infants with cholestasis at Fujian Maternity and Child Health Hospital from April 27, 2012, to July 11, 2023, were retrospectively analyzed as the development cohort. An external validation cohort was assembled from Fujian Children’s Hospital during the same period. Univariate analysis was initially conducted on the relevant indices, then the least absolute shrinkage and selection operator was performed to assess independent predictive factors. Further multivariate logistic regression analysis was conducted to identify independent predictors and develop predictive nomograms. Area under the curve (AUC) of receiver operating characteristic, calibration curves and decision curve analysis (DCA) were used to evaluate and validate the model and subsequently confirmed with the external validation group.

**Results:**

A total of 1,793 neonates and 583 infants were included in the development cohort, and 374 neonates and 232 infants in the external validation cohort. The neonatal nomogram included six variables that were significant independent predictors of LF: gestational age (*p* = 0.00), high-density lipoprotein (*p* = 0.008), red cell distribution width-standard deviation (*p* = 0.00), C-reactive protein (*p* = 0.00), albumin/fibrinogen (*p* = 0.00) and aspartate aminotransferase/platelets (*p* = 0.00). In the infant group, three variables, including vomiting (*p* = 0.005), lactate dehydrogenase (*p* = 0.00) and the albumin/fibrinogen (*p* = 0.00), were significant independent predictors of LF and were included in the infant nomogram. In the development cohort, the nomograms predicted LF with AUC values of 0.743 and 0.784 in the neonatal and infant groups, respectively. In the external validation cohort, the nomograms had AUC values of 0.736 and 0.711 in the neonatal and infant groups, respectively. The Hosmer–Lemeshow test results indicated that there was no significant difference between the predicted and true values. Calibration curves confirmed the consistency of the predicted outcomes with the real outcomes, and DCA curves demonstrated potential benefits for all patients.

**Conclusion:**

This study developed and externally validated age-specific models for predicting LF in cholestasis patients. These nomograms show good clinical utility and can help pediatricians identify LF cases early, potentially improving outcomes in Southeast China.

## Introduction

Cholestasis is identified by impaired bile flow from the liver to the duodenum, leading to the buildup of substances in the liver that are normally excreted into bile and eliminated through the intestines. International guideline indicates that approximately one in 2,500 full-term neonates are affected by neonatal cholestasis (Fawaz et al., 2017), However, there is a lack of relevant epidemiological data on the incidence of cholestasis in China (Subspecialty Group of Infectious Diseases, the Society of Pediatrics, Chinese Medical Association, 2022). In the evaluation of cholestasis, imaging methods play an important role. Abdominal ultrasound is fundamental for assessing biliary anatomy, gallbladder size and contractility, as well as liver texture (Rand, 2025). Hepatobiliary scintigraphy (HIDA scan) is frequently used to evaluate bile excretion and assist in the diagnosis of biliary atresia (Gregorio, Rey & Tan-Lim, 2025). Initial management of cholestasis includes the use of choleretic agents, supplementation with fat-soluble vitamins (A, D, E, and K), nutritional support with high-calorie formulas, and medium-chain triglyceride oil to improve absorption and nutritional status. Nevertheless, cholestasis can still lead to liver dysfunction and impaired nutrient absorption. It can also result in long-term complications such as liver fibrosis and cirrhosis, which may ultimately require liver transplantation and present a life-threatening emergency.

A study on infants with cholestasis revealed an overall mortality rate of 10.6% (425 out of 4,028) during the follow-up period, with 398 deaths (9.9%) occurring within the first year of life (Choi et al., 2022). Cholestasis clearly has a high mortality risk and poor prognosis; therefore, it is very important to predict the adverse consequences of cholestasis and make timely interventions. While imaging methods like abdominal ultrasound and HIDA scans are essential for identifying structural abnormalities and assessing bile excretion, they often do not provide early prognostic information about the progression to liver failure (LF). There are several prediction models for pediatric liver diseases, such as the Pediatric End-Stage Liver Disease score designed for chronic liver disease, the King’s College Hospital criteria score, and the Liver Injury Units utilized for acute LF (Freeman Jr et al., 2002; Liu et al., 2006; Jain & Dhawan, 2016), but the main goal of these models is to calculate the risk−benefit ratio associated with liver transplantation for young patients. As previously mentioned, the overall mortality rate among children with cholestasis is relatively high (10.6%) (Choi et al., 2022), by the time it becomes necessary to apply the aforementioned liver transplantation risk scoring criteria to determine whether these children require transplantation, their disease has often already progressed to a very severe stage. Therefore, there is a clear need to develop novel approaches for the early detection of adverse outcomes in children with cholestasis.

Patients with cholestasis often experience LF before death, and early detection of LF is therefore particularly important. However, limited research has been conducted on the predictors of cholestasis with LF. Moreover, the etiology of cholestasis in East Asian populations is distinct (Gottesman, Del Vecchio & Aronoff, 2015; Hahn et al., 2024; Kido et al., 2024), for example, cholestasis resulting from alpha-1-antitrypsin deficiency is comparatively prevalent among individuals of European and American ancestry, whereas it is exceedingly rare in East Asians. Conversely, citrin deficiency is more frequently observed in East Asians. In addition, the features of neonatal cholestasis also differ from those of infantile cholestasis. Therefore, the aim of this study was to identify predictors of cholestasis with LF in Southeast China and to use age stratification to develop two predictive models for neonates and infants separately. The ultimate goal is to enable early identification of cases of cholestasis with LF and treat them aggressively to avoid emergency liver transplantation and even death.

## Materials & Methods

### Study design and population

An analysis of clinical data obtained from the hospital database was conducted in this retrospective study. Patients aged 0–365 days who were admitted to Fujian Maternity and Child Health Hospital between April 27, 2012, and July 11, 2023, composed the development cohort. For validation purposes, we used data contributed by Fujian Children’s Hospital up until the specified deadline. The objective is to develop separate predictive models for LF in neonates and infants with cholestasis. Approval number 2020YJ229 was granted for the study by the ethics committee of Fujian Maternity and Child Health Hospital. Owing to the retrospective nature of the study, the ethics committee waived the requirement for informed consent from parents or caregivers.

The diagnostic criteria of cholestasis were as follows (Fawaz et al., 2017): direct/conjugated bilirubin levels >1.0 mg/dL (17mmol/L). The diagnostic criteria for LF were as follows (Wendon et al., 2017; Larson-Nath & Vitola, 2020; Borovsky et al., 2021; Mishra & Pallavi, 2022; Kelgeri et al., 2024): acute onset liver disease with no evidence of chronic liver disease, biochemical evidence of severe liver injury, coagulopathy that is not corrected by vitamin K: prothrombin time (PT) ≥15 s or international normalized ratio (INR) ≥1.5 with encephalopathy or PT ≥ 20 s or INR ≥ 2 with or without encephalopathy. Hepatic encephalopathy is be defined as brain dysfunction caused by liver insufficiency and/or portal-systemic shunting and manifests as a wide spectrum of neurological/psychiatric abnormalities ranging from subclinical alterations to coma (McLin & D’Antiga, 2022; Mishra & Pallavi, 2022; Bartlett & Kohli, 2024). Given that hepatic encephalopathy is sometimes difficult to define in neonates and infants, in the absence of clear evidence of hepatic encephalopathy, the diagnostic criterion of LF was defined as a PT ≥ 20 s or an INR ≥ 2.0 (Larson-Nath & Vitola, 2020). Patients aged 0–28 days of onset of cholestasis were classified into the neonatal group, whereas those aged 29–365 days of onset of cholestasis were classified into the infantile group. Patients with cholestasis who did not develop LF during the course of the disease were classified into the non-LF group, and those who developed LF during the course of the disease were classified into the LF group.

The inclusion criteria were as follows: (1) a confirmed diagnosis of cholestasis; (2) age at diagnosis of cholestasis less than 365 days; and 3) absence of LF at the time of cholestasis diagnosis.

The exclusion criteria were as follows: (1) patients with LF when cholestasis was diagnosed; (2) patients with missing data exceeding 25% (Dong et al., 2022); (3) patients with a gestational age of less than 28 weeks; (4) patients lacking coagulation function tests subsequent to the cholestasis diagnosis.

### Data collection

Experienced examiners thoroughly examined the comprehensive medical records of eligible patients to verify their eligibility for final inclusion. The following patient clinical characteristics and first laboratory results after onset of cholestasis were considered potential predictive variables and were extracted from the electronic information system. The demographic factors included the following: sex, onset age, gestational age, birth weight, mode of delivery and feeding. Clinical characteristics included fever, breathlessness, vomiting, abdominal distention, cyanosis, hepatomegaly, splenomegaly, intubation, congenital heart disease (CHD), and intracranial hemorrhage. Hepatomegaly is defined as the liver edge being palpable >3 cm below the right costal margin. Splenomegaly is defined as the spleen edge being palpable >1 cm below the left costal margin. The laboratory findings included routine blood tests, heart function tests, liver function tests, kidney function tests, electrolytes, and coagulation function tests.

### Statistical analysis

All computations were performed *via* R software (version 4.2.3). Details regarding data missingness are listed in Table S1, and missing data were imputed using multiple imputation as implemented by the “mice” package. The characteristics that were analysed were categorized based on age groups and described using proportions for categorical variables, whereas continuous variables were represented using means, standard deviations, medians, and interquartile ranges. Significant differences were evaluated *via* univariate analysis *via* the *χ*^2^ test for categorical variables and either Student’s t test or the Mann−Whitney U test for continuous variables, depending on the distributions and variances of the data. Least absolute shrinkage and selection operator (LASSO) regression analysis (*via* “glmnet”) was used in the development cohort to identify predictive factors. Further multivariate logistic regression analysis was conducted on the selected variables to identify independent predictors and develop a nomogram (*via* “rms”) for LF. The nomogram’s performance was assessed *via* the receiver operating characteristic (ROC) curve (*via* “pROC”), which measures the area under the ROC curve (AUC) ranging from 0.5 (indicating no ability to discriminate) to 1 (indicating full discriminatory ability). Additionally, Hosmer–Lemeshow tests and calibration curves (*via* “ResourceSelection” and “rms” packages) were used to compare the development and validation cohorts. Decision curve analysis (DCA, *via* “rmda” package) was conducted to establish the prediction’s net benefit threshold. “ggplot2″package was used for data visualization. Due to a substantial number of variables were incorporated in this study, and in order to adhere to biological plausibility and to identify variables strongly associated with LF, we applied a more stringent *p*-value threshold (*p* < 0.01) for variable selection.

## Results

### Case screening of cholestasis patients and cohort characteristics

**Figure 1:**
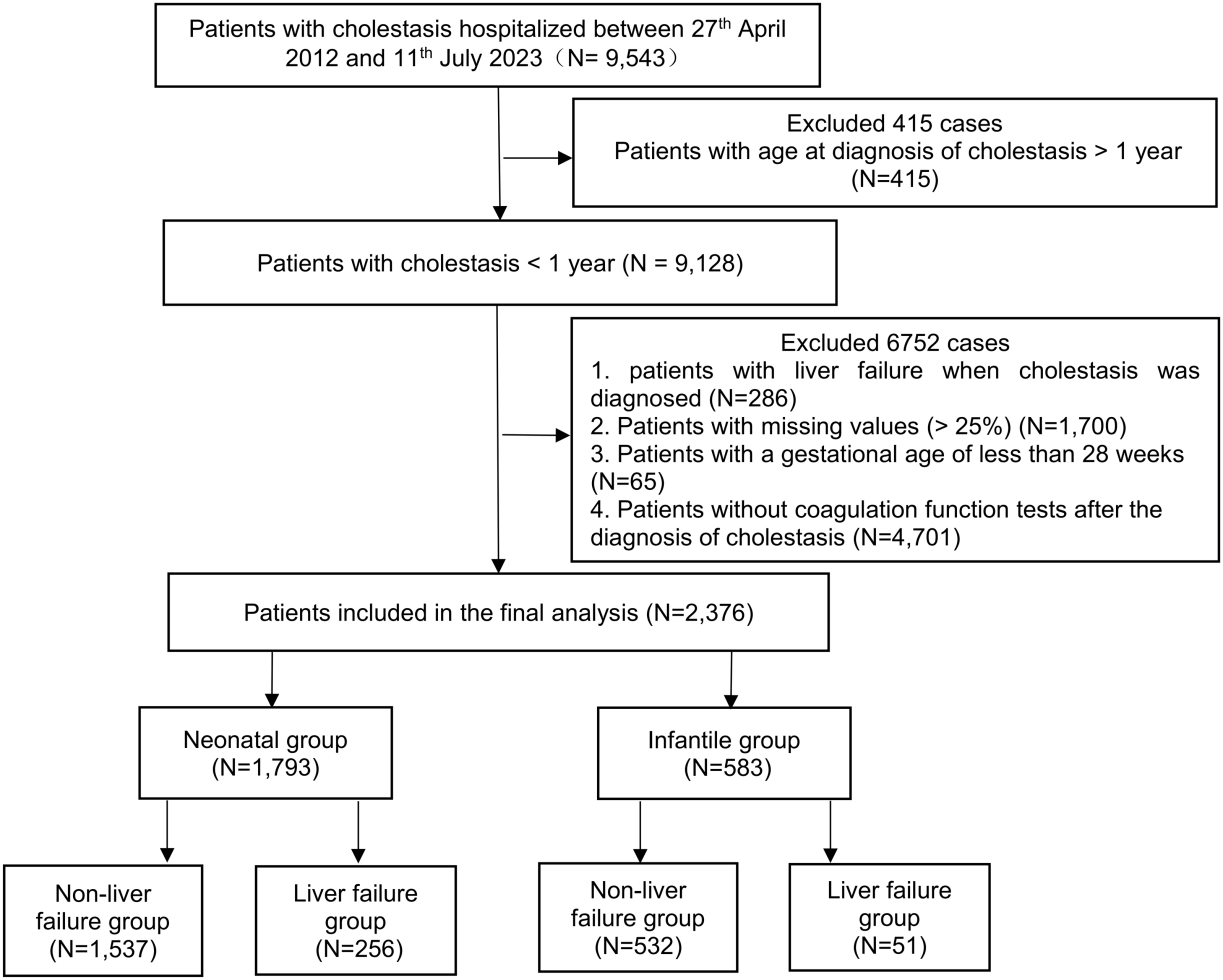
Flowchart detailing the screening and selection process for the development cohort. From 9,543 patients with cholestasis at Fujian Maternity and Child Health Hospital between April 27, 2012, and July 11, 2023, a total of 2,376 eligible patients with cholestasis were included. The final cohort was divided into a neonatal group (*n* = 1,793) and an infantile group (*n* = 583), which were then categorized based on the development of liver failure.

For the internal development cohort, 9,543 patients with cholestasis were examined at Fujian Maternity and Child Health Hospital. Following the elimination of 7,167 ineligible instances, 2,376 cases were incorporated into the final analysis as the development cohort (Fig. 1). Regarding the onset age distribution of cholestasis, 1,793 patients under the age of 28 days were classified into the neonatal development group. The median age was 5.07 days (interquartile range: 5.04–5.46), with a greater proportion of males (63.7%) than females (36.3%). Among them, 256 patients (14.3%) developed LF (LF group), whereas 1,537 patients (85.7%) did not develop LF (non-LF group). In the infantile development group, which consisted of 583 patients over the age of 29 days but under the age of 365 days, the median age was 50.56 days (interquartile range: 39.39–67.69). Similar to the neonatal group, there were more males (67.4%) than females (32.6%). Among these patients, 51 (8.7%) developed LF (LF group), whereas 532 patients (91.3%) did not develop LF (non-LF group).

For the external validation cohort, we included 606 patients from Fujian Children’s Hospital. Among these, 374 patients were classified into the neonatal group, with a median age of 5.20 days (interquartile range: 5.03–12.25). Once again, males (66.8%) represented a greater proportion than females did (33.2%). In this group, 55 patients (14.7%) developed LF (LF group), whereas 319 patients (85.3%) did not develop LF (non-LF group). Furthermore, the infantile group within the external validation cohort consisted of 232 patients. The median age in this group was 55.14 days (interquartile range: 42.51–74.66), with a greater proportion of males (62.9%) than females (37.1%). Among these patients, 17 (7.3%) developed LF (LF group), whereas 215 patients (92.7%) did not develop LF (non-LF group).

Tables S2 and S3 provide additional development and validation cohorts details.

### Diagnoses of the development and validation cohorts

The diagnoses of the cohorts primarily included structural abnormalities, infections, genetic/metabolic disorders, endocrine disorders, hematology and malignancy, cardiovascular disease, perinatal issues, drug-related factors, and idiopathic cholestasis.

In the neonatal group of the development cohort, there were 1,793 patients with overlapping diagnoses (*n* = 5,965). On average, each patient had 3.33 major diagnoses. In the validation cohort, 374 patients had overlapping diagnoses (*n* = 1,329). On average, each patient had 3.55 major diagnoses. The most common diagnosis in both cohorts was infection. In the development cohort, 65.70% of the patients were complicated with pneumonia compared to 60.43% of the patients in the validation cohort. Additionally, in the development cohort, 30.23% of the patients were complicated with sepsis compared to 26.74% of the patients in the validation cohort. Perinatal issues were the second most common diagnoses. In the development cohort, the most prevalent perinatal issues were gestational age <32 weeks (26.99%), birth asphyxia (26.49%), and birth weight <1,500 g (26.49%). In the validation cohort, the most common perinatal issues were gestational age <32 weeks (35.29%), birth weight <1,500 g (27.54%), and neonatal necrotizing enterocolitis (18.98%) (Table S4).

In the infantile group of the development cohort, 583 patients had overlapping diagnoses (*n* = 1,601). On average, each patient had 2.75 major diagnoses. In the validation cohort, there were 232 patients with overlapping diagnoses (*n* = 599). On average, each patient had 2.58 major diagnoses. Infection remained the most common diagnosis in both cohorts. In the development cohort, 40.99% of the patients were complicated with pneumonia compared to 33.62% of the patients in the validation cohort. Furthermore, 38.08% of the patients in the development cohort and 43.53% of the patients in the validation cohort were complicated with toxoplasma, other viruses, rubella virus, cytomegalovirus, and herpesvirus (TORCH) infections. Unlike the neonatal group, structural abnormalities were the second most common diagnoses in the infantile group. Among these, biliary atresia was found in 15.27% of the patients in the development cohort and in 18.10% of the patients in the validation cohort. Genetic/metabolic disorders were also significant diagnoses in the infantile group. Citrin protein deficiency was the most common genetic disease, affecting 10.29% of the patients in the development cohort and 6.47% of the patients in the validation cohort (Table S5).

### Demographics and clinical characteristics of the LF and non-LF groups in the development cohort

Among the neonatal participants, the non-LF group had a median onset age of 5.07 days (with an interquartile range of 5.04–5.49), whereas the LF group had a median onset age of 5.08 days (with an interquartile range of 5.04–5.31). Both groups had a greater proportion of males than females (63.2% *vs.* 36.8% and 66.8% *vs.* 33.2%). Compared with the non-LF group, the LF group had a greater percentage of patients whose gestational age was between 28–32 weeks (37.9% *vs.* 25.2%). Vomiting was more common in the non-LF group (7.3%) than in the LF group (3.9%). The LF group had a higher rate of intubation (25.0%) than did the non-LF group (15.5%). Additionally, the LF group had a greater incidence of CHD (44.1%) than did the non-LF group (34.4%). The most common CHDs in both groups were patent ductus arteriosus, atrial septal defects, and ventricular septal defects.

In the infantile group, the median onset age of the non-LF group was 49.89 days (interquartile range: 38.92–66.88), whereas the median onset age of the LF group was 57.99 days (interquartile range: 45.66–93.58). Similar to the neonatal group, there were more males than females in both the non-LF group (67.3% *vs.* 32.7%) and the LF group (68.6% *vs.* 31.4%). Fever and vomiting were more common in the LF group (31.4% and 21.6%) than in the non-LF group (15.4% and 7.9%, respectively). Compared with the non-LF group, the LF group had a greater rate of intubation (23.5% *vs.* 6.0%).

Following the univariate analysis in the development cohort, 25 variables were selected in the neonatal group, and 22 variables were selected in the infantile group (*P* < 0.01). Tables 1 and 2 present the demographics, clinical features, and laboratory results of the development cohort.

**Table 1:** Demographics and clinical characteristics among patients in the development cohort who did or did not develop LF.

**Characteristics**	**Neonatal group (*n* = 1, 793)**			**Infantile group (*n* = 583)**		
	**Non-LF (*n* = 1, 537)**	**LF (*n* = 256)**	***P***	**Non-LF (*n* = 532)**	**LF (*n* = 51)**	***P***
Sex, n (%)			0.27			0.85
Male	972 (63.2)	171 (66.8)		358 (67.3)	35 (68.6)	
Female	565 (36.8)	85 (33.2)		174 (32.7)	16 (31.4)	
Onset age (d), median (IQR)	5.07 (5.04, 5.49)	5.08 (5.04, 5.31)	0.45	49.89 (38.92, 66.88)	57.99 (45.66, 93.58)	0.007
Gestational age, n (%)			0.00			0.10
28–32 W	387 (25.2)	97 (37.9)		64 (12.0)	4 (7.8)	
32–37 W	486 (31.6)	67 (26.2)		67 (12.6)	2 (3.9)	
>37 W	664 (43.2)	92 (35.9)		401 (75.4)	45 (88.2)	
Birth weight, n (%)			0.18			0.55
<1,500 g	299 (19.5)	64 (25.0)		116 (21.8)	8 (15.7)	
1,500–2,500 g	528 (34.4)	88 (34.4)		106 (19.9)	8 (15.7)	
2,500–4,000 g	665 (43.3)	97 (37.9)		291 (54.7)	33 (64.7)	
>4,000 g	45 (2.9)	7 (2.7)		19 (3.6)	2 (3.9)	
Mode of delivery, n (%)			0.41			0.28
Cesarean delivery	1233 (80.2)	211 (82.4)		153 (28.8)	11 (21.6)	
Vaginal birth	304 (19.8)	45 (17.6)		379 (71.2)	40 (78.4)	
Feeding, n (%)			0.87			0.50
Breastfeeding	30 (2.0)	6 (2.3)		47 (8.8)	6 (11.8)	
Formula	1430 (93.0)	236 (92.2)		458 (86.1)	44 (86.3)	
Mixture	77 (5.0)	14 (5.5)		27 (5.1)	1 (2.0)	
Fever, n (%)			0.44			0.00
Yes	46 (3.0)	10 (3.9)		82 (15.4)	16 (31.4)	
No	1491 (97.0)	246 (96.1)		450 (84.6)	35 (68.6)	
Breathlessness, n (%)			0.06			0.83
Yes	950 (61.8)	174 (68.0)		79 (14.8)	7 (13.7)	
No	587 (38.2)	82 (32.0)		453 (85.2)	44 (86.3)	
Vomiting, n (%)			0.05			0.00
Yes	112 (7.3)	10 (3.9)		42 (7.9)	11 (21.6)	
No	1,425 (92.7)	246 (96.1)		490 (92.1)	40 (78.4)	
Abdominal distension, n (%)			0.51			0.96
Yes	75 (4.9)	15 (5.9)		35 (6.6)	4 (7.8)	
No	1,462 (95.1)	241 (94.1)		497 (93.4)	47 (92.2)	
Cyanosis, n (%)			0.10			0.87
Yes	179 (11.6)	39 (15.2)		43 (8.1)	5 (9.8)	
No	1,358 (88.4)	217 (84.8)		489 (91.9)	46 (90.2)	
Hepatomegaly, n (%)			0.29			0.42
Yes	1,017 (66.2)	178 (69.5)		323 (60.7)	28 (54.9)	
No	520 (33.8)	78 (30.5)		209 (39.3)	23 (45.1)	
Spleomegaly, n (%)			0.27			1.00
Yes	135 (8.8)	28 (10.9)		51 (9.6)	5 (9.8)	
No	1,402 (91.2)	228 (89.1)		481 (90.4)	46 (90.2)	
Intubation, n (%)			0.00			0.00
Yes	239 (15.5)	64 (25.0)		32 (6.0)	12 (23.5)	
No	1,298 (84.5)	192 (75.0)		500 (94.0)	39 (76.5)	
CHD, n (%)			0.00			0.10
Yes	529 (34.4)	113 (44.1)		42 (7.9)	8 (15.7)	
PDA	402 (26.2)	96 (37.5)		9 (1.7)	0 (0)	
ASD	70 (4.6)	9 (3.5)		12 (2.3)	1 (2)	
VSD	37 (2.4)	7 (2.7)		18 (3.4)	5 (9.8)	
Others	20 (1.3)	1 (0.4)		3 (0.6)	2 (3.9)	
No	1,008 (65.6)	143 (55.9)		490 (92.1)	43 (84.3)	
Intracranial hemorrhage, n (%)			0.25			0.16
Yes	101 (6.6)	12 (4.7)		23 (4.3)	5 (9.8)	
No	1,436 (93.4)	244 (95.3)		509 (95.7)	46 (90.2)	

**Notes.**

LF Liver faiure CHD Congenital heart disease PDA Patent ductus arteriosus ASD Atrial septal defect VSD Ventricular septal defect

**Table 2:** Laboratory findings among patients in the development cohort who did or did not develop LF.

Characteristics	Neonatal group (*n* = 1, 793)			Infantile group (*n* = 583)		
	Non-LF (*n* = 1, 537)	LF (*n* = 256)	*P*	Non-LF (*n* = 532)	LF (*n* = 51)	*P*
CMV infection, n (%)			0.39			0.03
Yes	71 (4.6)	15 (5.9)		196 (36.8)	11 (21.6)	
No	1,466 (95.4)	241 (94.1)		336 (63.2)	40 (78.4)	
Ca, median (IQR)	2.22 (2.09, 2.36)	2.20 (2.02, 2.33)	0.005	2.36 (2.23, 2.48)	2.25 (2.08, 2.40)	0.00
K, median (IQR)	4.40 (4.10, 4.80)	4.40 (3.90, 4.90)	0.46	5.00 (4.50, 5.50)	4.80 (4.40, 5.30)	0.11
Mg, median (IQR)	0.83 (0.75, 0.91)	0.84 (0.74, 0.94)	0.33	0.93 (0.87, 1.00)	0.91 (0.85, 1.00)	0.30
Na, median (IQR)	138.00 (136.00, 141.00)	138.00 (136.00, 140.00)	0.23	137.00 (135.00, 138.00)	136.00 (132.70, 138.50)	0.50
P, median (IQR)	1.82 (1.54, 2.11)	1.84 (1.43, 2.17)	0.45	1.81 (1.54, 2.01)	1.75 (1.35, 2.01)	0.34
ALP, median (IQR)	173.80 (132.40, 239.30)	196.25 (138.47, 270.42)	0.01	335.35 (252.55, 472.15)	301.80 (169.60, 515.40)	0.20
ALT, median (IQR)	9.40 (5.40, 16.20)	10.05 (5.48, 21)	0.04	49.20 (22.68, 98.63)	82.50 (23.75, 159.90)	0.08
AST, median (IQR)	30.80 (20.60, 48.30)	39.90 (21.70, 83.43)	0.00	66.00 (39.30, 115.53)	104.90 (46.50, 267.85)	0.007
DBil, median (IQR)	20.40 (19.10, 23.60)	20.80 (19.10, 23.93)	0.13	27.45 (19.30, 55.73)	31.80 (19.30, 57.80)	0.78
GGT, median (IQR)	138.10 (88.00, 224.80)	117.30 (65.65, 206.38)	0.005	142.35 (87.20, 263.60)	125.00 (61.90, 204.65)	0.04
IBil, median (IQR)	36.10 (22.80, 95.00)	35.20 (21.85, 60.33)	0.15	20.60 (10.60, 37.25)	23.00 (9.20, 36.45)	0.96
Prealbumin, median (IQR)	9.32 (7.73, 11.15)	8.23 (6.83, 9.89)	0.00	11.61 (8.66, 14.67)	8.86 (6.88, 12.35)	0.00
TBA, median (IQR)	10.40 (5.60, 16.85)	12.25 (6.40, 20.78)	0.02	47.57 (17.69, 79.75)	58.20 (35.50, 113.35)	0.02
TBil, median (IQR)	60.00 (45.00, 114.70)	60.44 (45.00, 91.28)	0.55	61.25 (36.70, 83.50)	57.00 (35.45, 95.30)	0.93
Total protein, median (IQR)	48.80 (44.10, 53.90)	46.55 (41.70, 53.18)	0.00	55.20 (51.00, 59.80)	50.60 (44.30, 58.20)	0.00
Globulin, median (IQR)	16.50 (13.70, 20.00)	16.20 (12.90, 20.03)	0.14	17.45 (14.98, 21.73)	15.50 (12.65, 21.40)	0.07
Cholesterol, median (IQR)	2.19 (1.70, 2.79)	1.94 (1.42, 2.58)	0.00	3.35 (2.70, 4.04)	2.80 (1.91, 3.93)	0.007
HDL, median (IQR)	0.84 (0.66, 1.07)	0.70 (0.50, 0.96)	0.00	0.91 (0.66, 1.24)	0.75 (0.46, 1.03)	0.00
LDL, median (IQR)	0.94 (0.61, 1.38)	0.88 (0.50, 1.31)	0.02	1.64 (1.18, 2.16)	1.54 (1.09, 1.81)	0.11
Triglyceride, median (IQR)	0.35 (0.20, 0.62)	0.35 (0.17, 0.67)	0.83	1.16 (0.71, 1.73)	1.05 (0.62, 1.75)	0.78
CK, median (IQR)	187.10 (96.00, 406.90)	212.40 (95.75, 459.45)	0.27	101.70 (69.10, 151.57)	126.90 (87.80, 240.40)	0.002
CKMB, median (IQR)	39.50 (25.80, 75.70)	46.45 (27.63, 121.38)	0.00	31.45 (23.30, 45.45)	43.00 (29.30, 81.70)	0.00
LDH, median (IQR)	415.00 (318.50, 559.90)	486.40 (316.62, 726.65)	0.00	320.06 (263.52, 408.60)	446.10 (284.15, 928.25)	0.00
Urea, median (IQR)	3.59 (2.58, 4.85)	3.64 (2.60, 5.04)	0.34	2.82 (2.03, 3.73)	3.00 (2.44, 6.29)	0.008
Creatinine, median (IQR)	55.90 (41.80, 71.50)	57.20 (41.55, 81.10)	0.06	28.15 (22.18, 41.58)	31.20 (21.65, 61.25)	0.25
Uric acid, median (IQR)	168.00 (100.00, 292.00)	170.00 (99.50, 313.00)	0.51	145.50 (110.00, 196.25)	200.00 (141.45, 370.00)	0.00
Leucocyte, median (IQR)	12.07 (9.10, 16.02)	11.64 (8.46, 16.74)	0.55	9.98 (7.83, 13.07)	11.45 (8.62, 16.54)	0.03
Neutrophil, median (IQR)	10.40 (9.70, 11.10)	10.60 (9.90, 11.50)	0.00	10.40 (9.70, 11.20)	10.30 (9.44, 11.14)	0.43
Lymphocyte, median (IQR)	4.17 (3.13, 5.36)	4.21 (3.19, 5.58)	0.41	5.35 (3.88, 7.17)	5.46 (3.45, 7.70)	0.91
Eosinophils, median (IQR)	0.36 (0.18, 0.62)	0.27 (0.14, 0.56)	0.00	0.28 (0.12, 0.50)	0.23 (0.10, 0.48)	0.68
Hematokrit, median (IQR)	42.30 (35.60, 49.40)	43.10 (33.55, 50.35)	0.69	30.70 (27.60, 34.00)	30.40 (27.43, 34.80)	0.71
Hemoglobin, median (IQR)	146.00 (123.00, 171.00)	143.00 (112.00, 173.50)	0.26	103.00 (91.00, 113.80)	103.00 (92.00, 114.50)	0.73
Erythrocyte, median (IQR)	4.31 (3.67, 4.96)	4.13 (3.47, 5.04)	0.15	3.46 (3.12, 3.85)	3.49 (3.03, 3.83)	0.91
MCHC, median (IQR)	346.00 (332.00, 357.00)	342.50 (328.00, 356.00)	0.02	336.00 (324.00, 348.00)	332.00 (322.00, 347.00)	0.57
RDWSD, median (IQR)	55.90 (50.90, 61.50)	58.39 (52.19, 65.47)	0.00	48.53 (44.27, 53.50)	50.90 (45.76, 57.49)	0.04
MCV, median (IQR)	99.00 (93.60 104.50)	99.45 (93.80, 106.93)	0.14	88.85 (85.20, 93.10)	87.40 (84.30, 92.20)	0.23
PLT, median (IQR)	272.00 (194.00, 359.00)	207.50 (136.75, 311.50)	0.00	391.00 (277.25, 499.00)	326.00 (147.00, 449.50)	0.00
CRP, median (IQR)	0.50 (0.50, 1.87)	1.02 (0.50, 4.64)	0.00	0.77 (0.50, 3.73)	2.73 (1.21, 15.29)	0.00
Blood glugose, median (IQR)	3.78 (2.80, 4.78)	3.53 (2.38, 4.69)	0.13	4.66 (3.95, 5.62)	4.90 (3.74, 5.94)	0.35
Ddimer, median (IQR)	2.01 (0.93, 4.42)	2.48 (1.15, 5.67)	0.005	1.04 (0.51, 2.14)	2.14 (0.72, 5.10)	0.01
GGT/Albumin, median (IQR)	4.23 (2.72, 7.08)	3.65 (2.30, 6.70)	0.05	3.93 (2.25, 7.35)	3.78 (1.91, 6.22)	0.22
GGT/ALT, median (IQR)	15.06 (7.52, 29.01)	12.11 (4.21, 25.12)	0.00	3.37 (1.53, 6.48)	1.98 (0.68, 3.97)	0.00
Triglyceride/HDL, median (IQR)	0.42 (0.21, 0.84)	0.54 (0.23, 1.31)	0.007	1.29 (0.64, 2.35)	1.73 (0.70, 3.16)	0.09
Uric acid/Creatinine, median (IQR)	0.03 (0.01, 0.09)	0.05 (0.01, 0.20)	0.00	4.95 (3.15, 7.39)	6.61 (3.88, 10.29)	0.00
ALP/Albumin, median (IQR)	0.07 (0.03, 0.13)	0.08 (0.04, 0.24)	0.00	9.26 (6.73, 12.45)	8.70 (5.11, 14.69)	0.57
PLT/Lymphocyte, median (IQR)	66.83 (46.40, 92.14)	50.42 (32.7, 77.67)	0.00	70.36 (46.55, 101.58)	48.16 (30.11, 87.01)	0.00
Neutrophil/Lymphocyte, median (IQR)	2.51 (1.95, 3.27)	2.53 (1.84, 3.39)	0.94	0.51 (0.38, 0.70)	0.53 (0.31, 0.77)	0.94
Albumin/Fib, median (IQR)	17.86 (12.82, 23.50)	20.90 (13.45, 30.56)	0.00	17.03 (13.51, 22.72)	22.32 (14.12, 40.59)	0.00
AST/PLT, median (IQR)	0.12 (0.066, 0.22)	0.20 (0.08, 0.49)	0.00	0.17 (0.10, 0.36)	0.30 (0.12, 2.16)	0.00
Lymphocyte/leucocyte, median (IQR)	0.36 (0.25, 0.47)	0.38 (0.27, 0.50)	0.08	0.57 (0.45, 0.67)	0.51 (0.33, 0.66)	0.03

**Notes.**

LF Liver faiure CMV Cytomegalovirus Ca Calcium K Kalium Mg Magnesium Na Natrium P Phosphorus ALP Alkaline phosphatase ALT Alanine aminotransferase AST Aspartate aminotransferase DBil Direct bilirubin GGT Gamma-glutamyl transpeptidase IBil Indirect bilirubin TBA Total bile acid TBil Total bilirubin HDL High density lipoprotein LDL Low density lipoprotein CK Creatine kinase CKMB Creatine kinase-MB LDH Lactate dehydrogenase MCHC Mean corpuscular hemoglobin concentration RDWSD Red cell distribution width-standard deviation MCV Mean corpuscular volume PLT Platelet CRP C-reactive protein Fib Fibrinogen

### LASSO regression for variable screening

To address concerns of overfitting and potential collinearity among variables measured from the same patient, LASSO regression was utilized. The value of *λ* min determines the extent to which the sizes of the coefficients in a regression model are penalized. By applying stronger penalties, estimates associated with weaker factors tend to shrink towards zero, allowing only the most robust predictors to remain in the model. *λ* min was used to identify the covariates with the highest predictive power.

**Figure 2:**
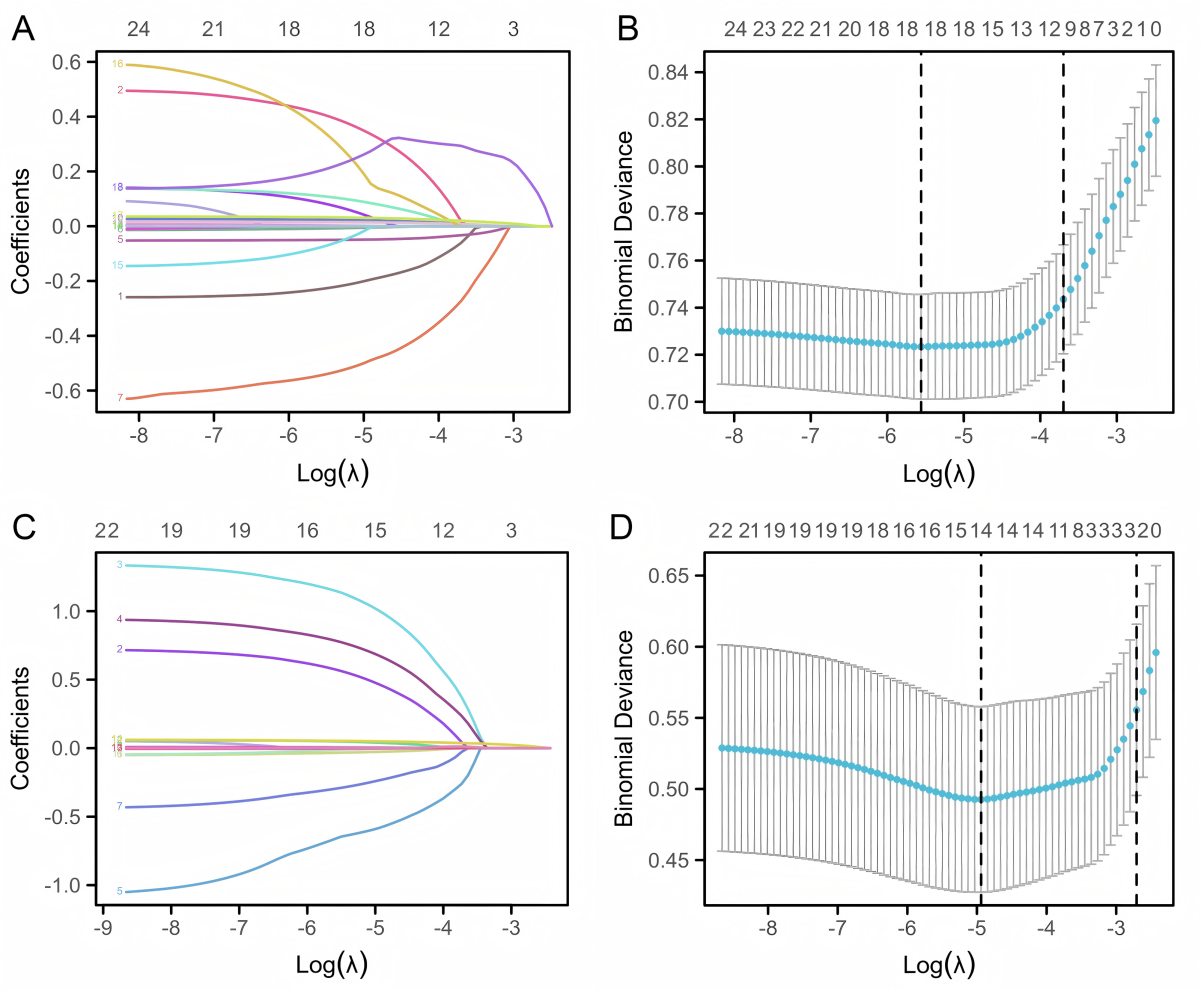
LASSO regression for screening predictive variables of LF among cholestasis patients. (A) Coefficient profiles of the 25 candidate variables for predicting LF in the neonatal group; each curve represents one variable, showing how its coefficient changes with the tuning parameter (*λ*), the 18 selected variables were numbered. (B) 10-fold cross-validation for tuning parameter (*λ*) selection in the neonatal group , which resulted in the selection of 18 variables with non-zero coefficients for further analysis. (C) Coefficient profiles of the 22 candidate variables for the infantile group, the 14 selected variables were numbered. (D) 10-fold cross-validation for tuning parameter (*λ*) selection in the neonatal group, which resulted in the selection of 14 variables with non-zero coefficients for further analysis. The numbers on the *X*-axis at the top of A and C represent the number of variables with non-zero coefficients at each *λ* value. LASSO, Least absolute shrinkage and selection operator; LF, Liver failure.

Following the LASSO regression selection process, 18 variables were identified as significant predictors of LF within the neonatal group. These variables included gestational age, intubation, CHD, aspartate aminotransferase (AST), prealbumin, total protein, high-density lipoprotein (HDL), creatine kinase-MB (CKMB), neutrophil count, red cell distribution width-standard deviation (RDWSD), platelet count (PLT), C-reactive protein (CRP), Ddimer, gamma-glutamyl transpeptidase (GGT)/alanine aminotransferase (ALT), uric acid/creatinine, alkaline phosphatase (ALP)/albumin, albumin/fibrinogen (Fib) and AST/PLT (Figs. 2A–2B).

Similarly, within the infantile group, 14 variables remained significant predictors of LF. These variables included onset age, fever, vomiting, intubation, calcium (Ca), prealbumin, HDL, CKMB, lactate dehydrogenase (LDH), uric acid, GGT/ALT, uric acid/creatinine, PLT/lymphocyte and albumin/Fib (Figs. 2C–2D).

### Logistic regression for building the prediction model

Within the neonatal group, the inclusion of the aforementioned 18 variables in a logistic regression model revealed six significant independent predictors of LF, and these were subsequently incorporated into the risk score. These variables consisted of gestational age, HDL, RDWSD, CRP, the albumin/Fib ratio, and the AST/PLT ratio (Table 3).

Similarly, for the infantile group, the logistic regression model incorporating the aforementioned 14 variables identified three significant independent predictors of LF, which were included in the risk score. These predictors included vomiting, LDH, and the albumin/Fib ratio (Table 4).

**Figure 3:**
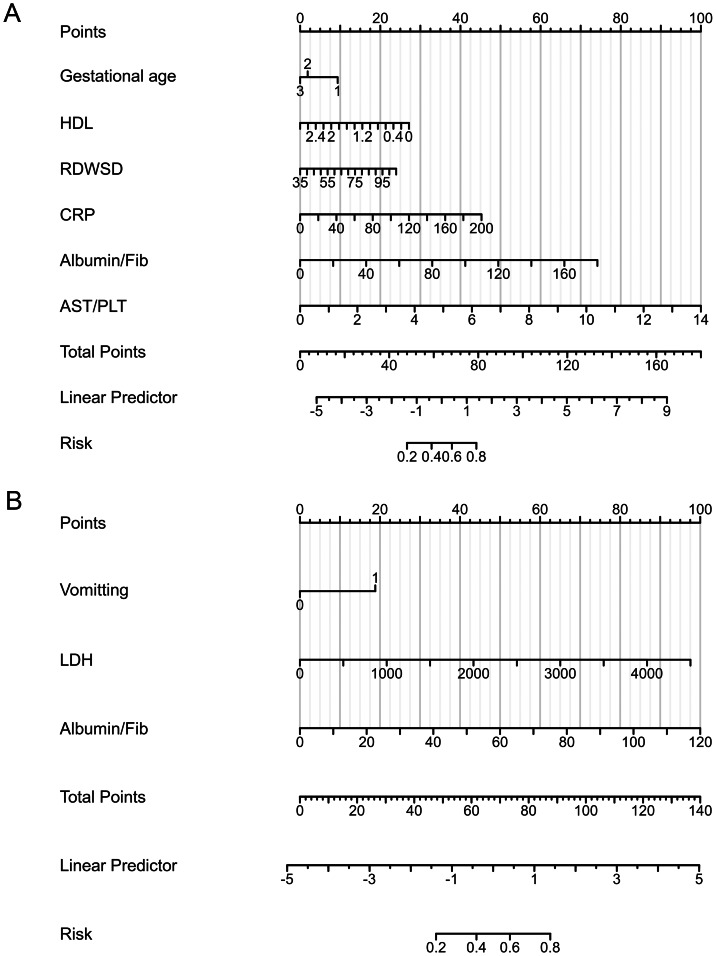
Nomogram prediction model for LF among cholestasis patients. (A) Nomogram prediction model for LF of the neonatal group. For gestational age, 1 indicates 28–32 weeks, 2 indicates 32–37 weeks, and 3 indicates >37 weeks. (B) Nomogram prediction model for LF of the infantile group. For Vomiting, 1 indicates Yes and 2 indicates No. To use: For each patient, locate the value for each predictor on its corresponding axis, draw a line upward to determine points, sum the points, and locate the total on the “Total Points” axis to estimate risk of LF. LF, Liver failure; HDL, High density lipoprotein; RDWSD, Red cell distribution width-standard deviation; CRP, C-reactive protein; Fib, Fibrinogen; AST, Aspartate aminotransferase; PLT, Platelet; LDH, Lactate dehydrogenase.

The final logistic models were developed as nomograms, with the neonatal nomogram presented in Fig. 3A and the infantile nomogram presented in Fig. 3B.

### Validation of the LF prediction model

**Figure 4:**
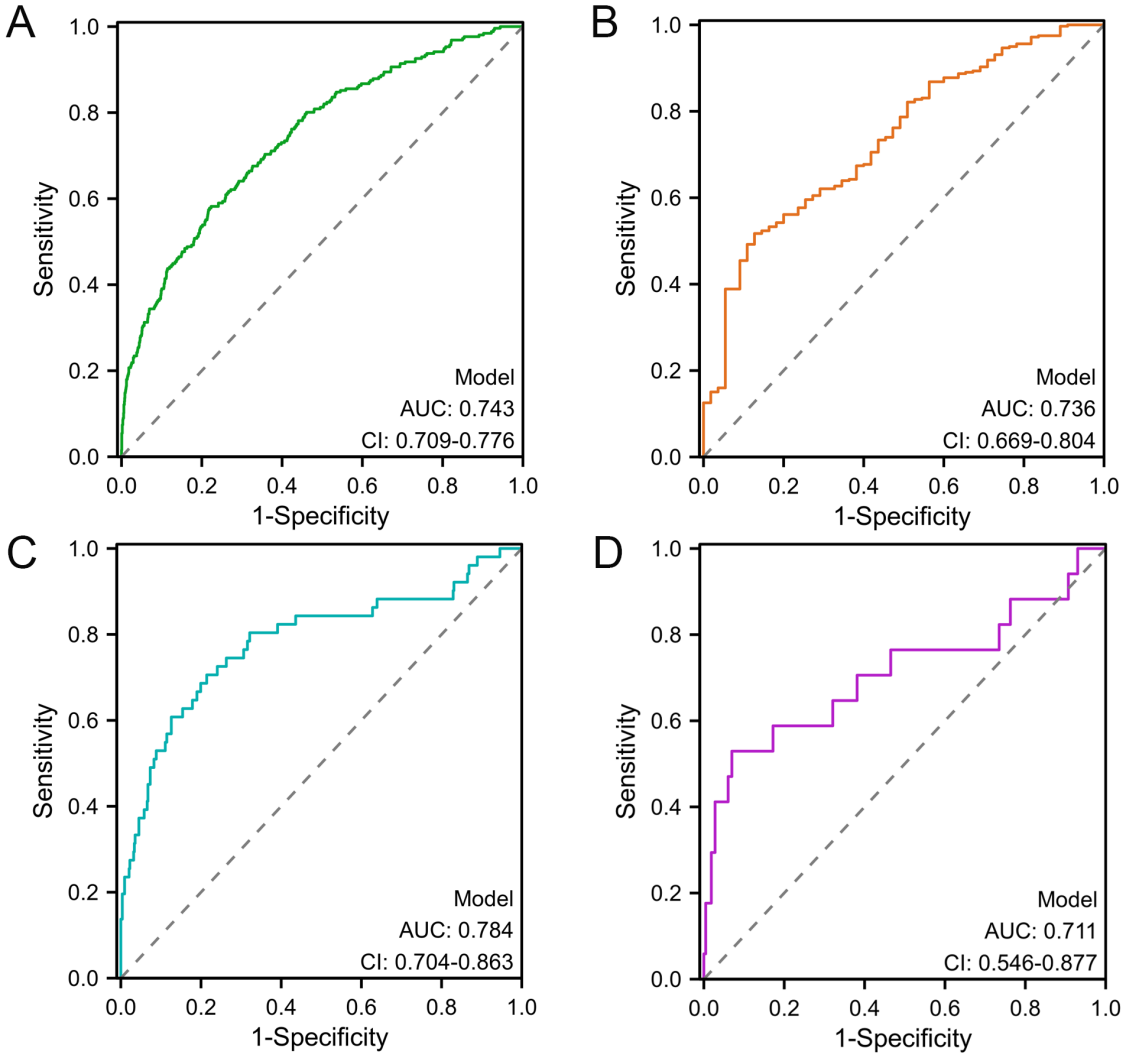
ROC analysis of the nomogram for predicting LF among cholestasis patients. (A) ROC curve of the nomogram prediction model in the neonatal development cohort. (B) ROC curve of the nomogram prediction model in the neonatal validation cohort. (C) ROC curve of the nomogram prediction model in the infantile development cohort. (D) ROC curve of the nomogram prediction model in the infantile validation cohort. ROC, Receiver operating characteristic; LF, liver failure.

The AUCs for the development neonatal and infantile groups were 0.743 (95% confidence interval (CI) [0.709–0.776]) and 0.784 (95% CI [0.704–0.863]), respectively (Figs. 4A, 4C). In the external validation cohort, the AUCs for the neonatal and infantile groups were 0.736 (95% CI [0.669–0.804]) and 0.711 (95% CI [0.546–0.877]), respectively (Figs. 4B, 4D).

**Figure 5:**
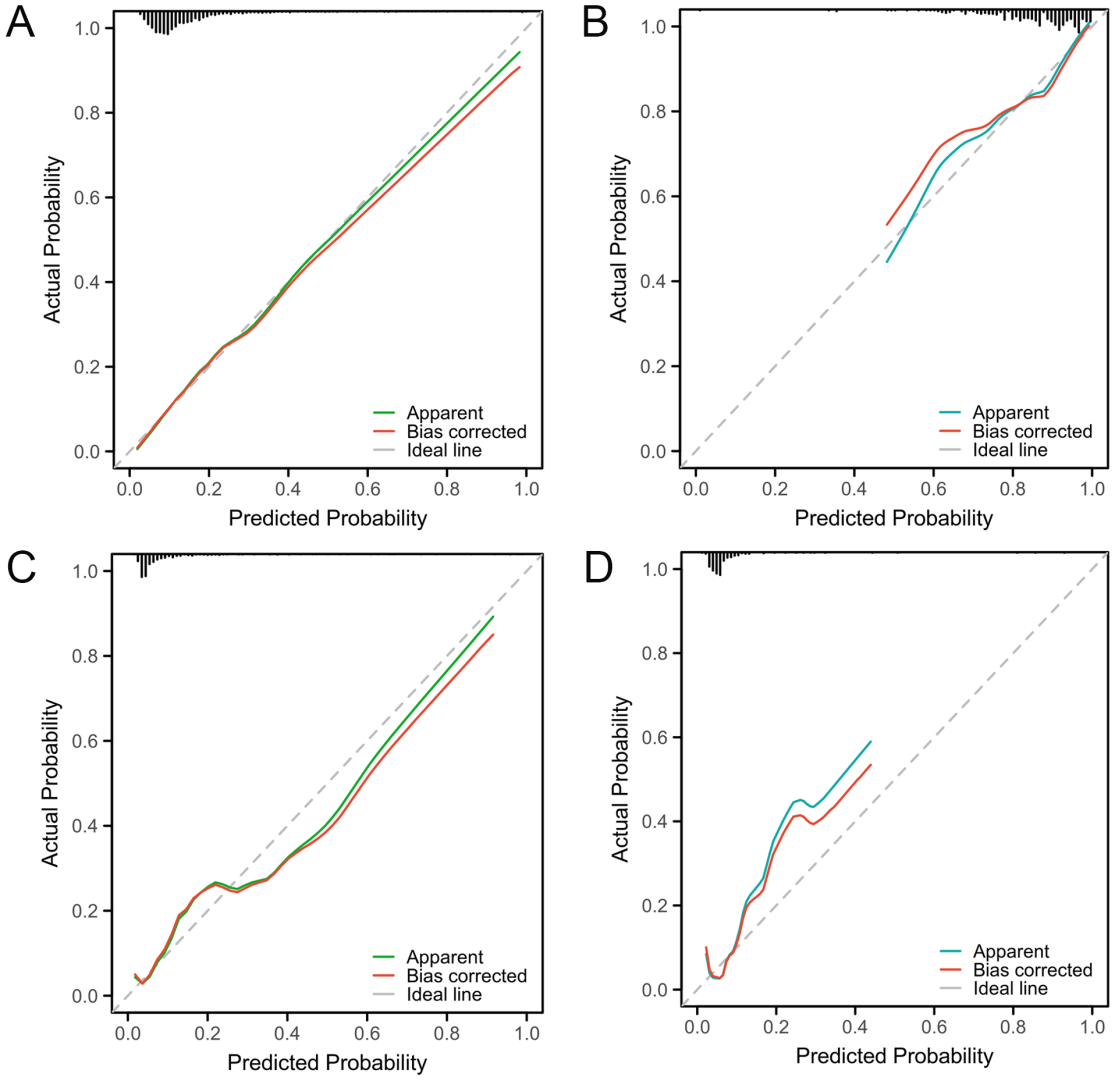
Calibration curves for nomogram models predicting LF among cholestasis patients. (A) Calibration curves of the nomogram prediction model in the neonatal development cohort. (B) Calibration curves of the nomogram prediction model in the neonatal validation cohort. (C) Calibration curves of the nomogram prediction model in the infantile development cohort. (D) Calibration curves of the nomogram prediction model in the infantile validation cohort. The calibration curve is used to assess the agreement between the model’s predicted outcomes and the actual outcomes. The apparent curve represents the predicted curve, the bias corrected curve represents the calibration curve, and the ideal line represents the reference (perfect) curve. The closer the calibration curve is to the ideal line, the better the predictive performance of the model. LF, Liver failure.

The Hosmer–Lemeshow test results in the neonatal development cohort and the infantile development cohort indicated no significant difference between the predicted and observed values (*P* = 0.91 and *P* = 0.15, respectively). The results indicated a strong correspondence between the predicted outcomes and the observed findings. The calibration curve is used to assess the agreement between the model’s predicted outcomes and the actual outcomes. The calibration curves of these models in both development and validation cohorts closely resembled the ideal curve, further confirming the consistency between the predicted and actual outcomes. The calibration curves for both the development and validation cohorts are depicted in Figs. 5A–5D.

**Figure 6:**
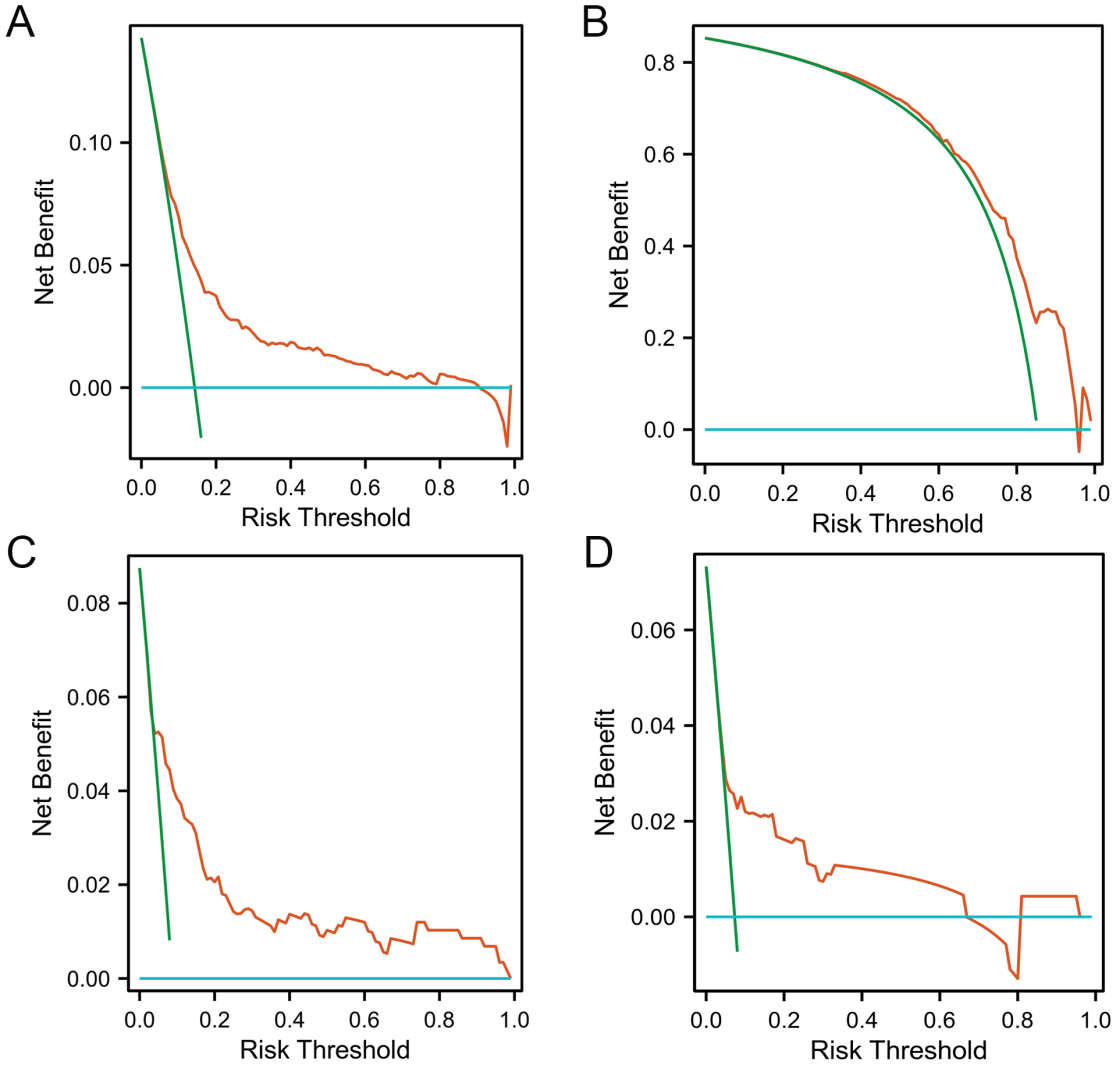
DCA of the nomogram model s for predicting LF among cholestasis patients. (A) DCA of the nomogram model in the neonatal development cohort. (B) DCA of the nomogram model in the neonatal validation cohort. (C) DCA of the nomogram model in the infantile development cohort. (D) DCA of the nomogram model in the infantile validation cohort. DCA evaluates the clinical utility of the nomogram models by quantifying the net benefit at different risk thresholds. The *Y*-axis represents the net benefit, and the *X*-axis represents the risk threshold, which is the probability threshold at which a clinician might decide to intervene. The red line (model) represents the net benefit of using the nomogram to make decisions, the green line (“all treatment line”) represents the net benefit of treating all patients, and the blue line (“no treatment line”) represents the net benefit of treating no patients. The greater area between the red line and the blue (“no treatment line”) and green line (“all treatment line”) lines indicates better clinical applications of the predictive models of LF. DCA, Decision curve analysis; LF, Liver failure.

Furthermore, we conducted DCA to assess the clinical application value of the nomograms in predicting LF. The greater area between the red line and the blue (“no treatment line”) and green line (“all treatment line”) lines indicates better clinical applications of the predictive models of LF. In the neonatal model, when the probability threshold of the development cohort was 0.18–0.90, the net benefit of the model was greater, and when the probability threshold of the validation cohort was 0.40–0.98, the net benefit of the model was greater (Figs. 6A–6B). In the infantile model, when the probability threshold of the development cohort was 0.05–1.00, the net benefit of the model was greater, and when the probability threshold of the validation cohort was 0.04–0.95, the net benefit of the model was greater (Figs. 6C–6D).

**Table 3:** Multivariable logistic regression model for predicting the development of LF in the neonatal group.

**Variables**	**Wald**	***P***	**OR**
Gestational age	9.205	0.00	0.746
HDL	7.026	0.008	0.509
RDWSD	11.719	0.00	1.028
CRP	20.042	0.00	1.018
Albumin/Fib	42.614	0.00	1.037
AST/PLT	12.949	0.00	1.647
Constant	17.661	0.00	0.012

**Notes.**

LF Liver faiure HDL High density lipoprotein RDWSD Red cell distribution width-standard deviation CRP C-reactive protein Fib Fibrinogen AST Aspartate aminotransferase PLT Platelet

**Table 4:** Multivariable logistic regression model for predicting the development of LF in the infantile group.

**Variables**	**Wald**	***P***	**OR**
Vomiting	7.903	0.005	3.290
LDH	16.660	0.00	1.001
Albumin/Fib	26.605	0.00	1.062
Constant	0.900	0.34	0.225

**Notes.**

LF Liver faiure LDH Lactate dehydrogenase Fib Fibrinogen

## Discussion

This study examined predictors of LF in critically ill children with cholestasis. As shown in the results section, the etiological differences between neonates and infants and the resulting differences in clinical presentation were key factors in our decision to use age stratification. We developed models for neonates and infants separately for the early prediction of cholestasis with LF in Southeast China. The neonatal model included six variables, whereas the infantile model included three variables. The results from the external cohorts confirmed that the prediction model exhibited excellent discriminability, consistency and validity. These models are expected to allow pediatricians to identify severe cases of cholestasis that are about to develop LF at an early stage and treat them aggressively to avoid emergency liver transplantation and even death.

In this study, neonates and infants with LF often had multiple underlying causes, with each patient typically having more than two major diagnoses. This overlap indicates that early-life LF commonly results from a combination of factors, increasing the complexity of diagnosis and treatment. In the neonatal group, infection was the most common etiology. Due to immature immune and liver function, neonates are particularly susceptible to perinatal infections and hypoxic stressors, with pneumonia, sepsis, and perinatal complications constituting the primary etiologies (Tang & Tang, 2021). For infants, although infection remained most common, pneumonia and TORCH infections accounted for a higher proportion. This may be because congenital or perinatally acquired TORCH infections become clinically apparent, or are more frequently recognized during infancy, resulting in distinct differences in etiology between these two groups (Horlenko et al., 2022).

With respect to demographics and clinical characteristics, studies from both China and other regions have consistently reported a higher proportion of males with cholestasis (Dong et al., 2018; Zhao et al., 2021; Choi et al., 2022). In our study, males outnumbered females in both the neonatal and infant groups (approximately 2:1), the ratio being consistent with previous findings (Gürcan Kaya et al., 2025). The underlying reasons for this sex difference remain unclear. It has been suggested that androgens may make males more prone to liver injury (Stimac et al., 2002), and whether genetic or sex chromosome factors contribute to the increased susceptibility to cholestasis in males remains to be elucidated and warrants further investigation. Gestational age was identified as a predictor of LF in the neonatal group. Research has shown that small for gestational age (SGA) is a significant and separate factor that increases the risk of neonatal cholestasis (Champion et al., 2012). Compared with the non-LF group, the LF group presented a greater percentage of patients with SGA. Since the etiology of infantile cholestasis differs from that observed in neonates, and SGA does not constitute a primary contributor to its pathogenesis, gestational age accordingly lacks predictive value for infantile LF. In our study, vomiting was the only symptom found to be significantly associated with infantile LF during the early stages of LF. Vomiting can result from the impaired ability of the liver to process toxins, drugs, or waste products, leading to their accumulation in the body. Furthermore, LF can lead to complications such as gastrointestinal bleeding, hyperammonemia (Duarte et al., 2023), and metabolic acidosis, which can all lead to vomiting. Therefore, when cholestatic infants exhibit vomiting not attributed to gastroenteritis, vigilance towards the possibility of LF is necessary. However, the incidence of vomiting in neonates with LF was lower than in those without LF. This may be due to the more critical condition of these patients, as impaired consciousness and the use of sedative medications can suppress the vomiting reflex. In addition, attention in severe cases may be focused on more urgent symptoms, leading to underreporting of vomiting. Mild vomiting might also be mistaken for physiological regurgitation. Finally, the non-LF group may include more cases with gastrointestinal malformations, infections, or metabolic diseases, which could contribute to the higher rate of vomiting observed.

With respect to laboratory findings, the same factors, the albumin/Fib ratio, was used to predict LF in both the neonatal and infantile groups. Both albumin and Fib are synthesized in the liver, and their synthesis is reduced when liver function is impaired. Our study revealed that the LF group presented a greater albumin/Fib ratio than did the non-LF group. However, another study suggested that a high Fib−albumin ratio (>7.6%) was significantly associated with decreased overall survival or disease-free survival (*p* = 0.00). These findings indicate that elevated Fib/albumin levels are related to adverse outcomes, which differs from the findings of our study. One possible explanation is that LF, which was the outcome measure in our study, is characterized primarily by significant coagulation disorders. Once LF occurs, the synthesis of coagulation factors is reduced, leading to prolonged PT, increased INR, and a significant decrease in Fib levels (Shao et al., 2015). The decrease in Fib is greater than the decrease in albumin, resulting in an increase in the albumin/Fib ratio.

In the neonatal group, HDL, RDWSD, CRP and AST/PLT were unique independent predictors of LF. Studies have shown that, in acute and acute-on-chronic LF, HDL levels are significantly lower in non-survivors (Etogo-Asse et al., 2012; Trieb et al., 2020), which is consistent with our results. HDL can exert protective effects by promoting the production of nitric oxide, inhibiting inflammatory factors such as TNF-*α* and IL-6, and enhancing the activity of antioxidant enzymes (Das & Ingole, 2023; Zhang et al., 2024). Therefore, improvement of HDL function can alleviate hepatic inflammation and promote hepatocyte repair. The significant reduction of HDL suggests its potential as a predictor of LF in neonatal cholestasis. A study revealed that an elevation in the red cell distribution width (RDW) at admission increased the risk of transplant or mortality in LF patients (Caire & Stravitz, 2012). Another study revealed that RDW serves as a separate predictive marker for death in individuals with acute-on-chronic LF associated with hepatitis B virus (Qin et al., 2018). Therefore, RDW can serve as a predictive and prognostic marker for hepatic injury and liver disease. CRP, a liver-produced protein, was found to play a role in predicting LF. On the one hand, LF leads to increased inflammation in the body, resulting in elevated CRP levels. On the other hand, infection is the primary cause of neonatal LF; the higher the CRP level is, the more severe the infection is likely to be and the greater the risk of LF. When the liver is damaged, AST levels increase. Liver dysfunction can cause decreased PLT production or increased PLT consumption, resulting in reduced PLT levels. AST/PLT has previously been used as an indicator for evaluating liver fibrosis (Yan, Xiao & Yang, 2015; Dhananjay & Pulloori, 2021). A higher AST/PLT ratio suggests more severe liver disease. For the first time, we found that the AST/PLT ratio was an independent predictor of neonatal LF.

In the infantile group, LDH was as independent predictor of LF. LDH increases under low oxygen concentrations, and hepatic hypoxic conditions exist in acute LF, resulting in increased production of LDH in hepatocytes (Kotoh et al., 2011). In experimental mouse models (Ferriero et al., 2018), LDH relocates to the nucleus, resulting in harmful gene expression. The administration of an LDH inhibitor has been shown to decrease liver damage and increase survival. LDH has been identified as a predictor of mortality in patients with acetaminophen-induced acute LF (Vazquez et al., 2022). Consistent with these studies, our findings demonstrated a significant increase in LDH in LF.

There are a few limitations that need to be addressed in our study. First, this retrospective study included only patients hospitalized in two centers during a specific period with relatively complete medical records, which may not represent the entire target population. Additionally, indicators such as vomiting in neonates may have been incompletely or inconsistently recorded, potentially introducing certain biases. Second, some important indicators, such as coagulation function, had many missing values, making it impossible to include them in the analysis and resulting in the exclusion of many cases. This likely introduced a selection bias, as both the most critically ill patients (who may have been too unstable for testing or died prior to testing, though these cases were relatively few) and the least ill patients (for whom testing was deemed unnecessary, comprising the majority) were systematically excluded. However, as our sample size was relatively large, we think this had limited impact on the results. To ensure data collection for most patients, our analysis primarily incorporated regular elements from medical records, nevertheless, some data were still missing and required imputation, which may have introduced some biases. Third, the inclusion of a relatively large number of variables (six variables) in the neonatal nomogram may decrease the value of the study. Finally, the current study did not assess the influence of clinical management; therefore, no inferences can be made about the correlation between therapeutic approaches and the progression of the disease.

## Conclusions

Our study is the first to develop and externally validate age-specific forecasting nomograms for LF in neonatal and infantile cholestasis. These nomograms demonstrated good predictive performance and clinical utility, which may enable pediatricians to more accurately identify LF in children with cholestasis at an early stage. Their application could contribute to timely intervention and improved prognoses for affected patients with cholestasis in Southeast China.

## Supplemental Information

10.7717/peerj.20800/supp-1 Supplemental Information 1 The distribution of missing data for variables in the development and validation cohorts

10.7717/peerj.20800/supp-2 Supplemental Information 2 Demographics and clinical characteristics between development cohort and validation cohort LF: Liver faiure; CHD: Congenital heart disease ; PDA: Patent ductus arteriosus; ASD: Atrial septal defect; VSD: Ventricular septal defect.

10.7717/peerj.20800/supp-3 Supplemental Information 3 Laboratory Findings between development cohort and validation cohort CMV: Cytomegalovirus; Ca: Calcium; K: Kalium; Mg: Magnesium; Na: Natrium; P: Phosphorus; ALP: Alkaline phosphatase; ALT: Alanine aminotransferase; AST: Aspartate aminotransferase; DBil: Direct dilirubin; GGT: Gamma-glutamyl transpeptidase; IBil: Indirect bilirubin; TBA: Total bile acid; TBil: Total bilirubin; HDL: High density lipoprotein; LDL: Low density lipoprotein; CK: Creatine kinase; CKMB: Creatine kinase-MB; LDH: Lactate dehydrogenase; MCHC: Mean corpuscular haemoglobin concentration; RDWSD: Red cell distribution width- standard deviation; MCV: Mean corpuscular volume; PLT: Platelet; CRP: C-reactive protein ; Fib: Fibrinogen.

10.7717/peerj.20800/supp-4 Supplemental Information 4 The diagnoses of neonatal group There were 1793 patients in the development cohort, overlapped diagnoses (n = 5965 ) with a mean of 3.33 major health issues (or diagnoses) were noted per patient. There were 374 patients in the validation cohort, overlapped diagnoses (n = 1329 ) with a mean of 3.55 major health issues (or diagnoses) were noted per patient. TORCH: Toxoplasma, Others, Rubella virus, Cytomegalovirus, Herpes virus; PFIC: Progressive familial intrahepatic cholestasis; NTCP: Sodium taurocholate cotransporting polypeptide; NEC: Neonatal necrotizing enterocolitis.

10.7717/peerj.20800/supp-5 Supplemental Information 5 The diagnoses of infantile group There were 583 patients in the development cohort, overlapped diagnoses (n = 1601) with a mean of 2.75 major health issues (or diagnoses) were noted per patient. There were 232 patients in the validation cohort , overlapped diagnoses (n = 599) with a mean of 2.58 major health issues (or diagnoses) were noted per patient. TORCH: Toxoplasma, Others, Rubella virus, Cytomegalovirus, Herpes virus; PFIC: Progressive familial intrahepatic cholestasis; NTCP: Sodium taurocholate cotransporting polypeptide; NEC: Neonatal necrotizing enterocolitis .

10.7717/peerj.20800/supp-6 Supplemental Information 6 The neonatal development cohort

10.7717/peerj.20800/supp-7 Supplemental Information 7 The neonatal validation cohort

10.7717/peerj.20800/supp-8 Supplemental Information 8 The infantile development cohort

10.7717/peerj.20800/supp-9 Supplemental Information 9 The infantile validation cohort

## Additional Information and Declarations

### Data Availability

The following information was supplied regarding data availability: The raw data are available in the Supplementary Files.
