# Supplementary material for: Retrospective assessment of the predictors of neonatal and infantile cholestasis with and without liver failure: an experience from Southeast China
Source: PeerJ. 2026 Feb 10;14:e20800. doi: 10.7717/peerj.20800 (PMC12903896; doi:10.7717/peerj.20800)
Supplement: Supplemental Information 2 — LF: Liver faiure; CHD: Congenital heart disease ; PDA: Patent ductus arteriosus; ASD: Atrial septal defect; VSD: Ventricular septal defect. [file peerj-14-20800-s002.docx]

| **Characteristics** | **Neonatal group** | | **Infantile group** | |
| --- | --- | --- | --- | --- |
|  | **Development cohort (n=1793)** | **Validation cohort (n=374)** | **Development cohort (n=583)** | **Validation cohort (n=232)** |
| LF, n (%) |  |  |  |  |
| Yes | 256 (14.3) | 55 (14.7) | 51 (8.7) | 17 (7.3) |
| No | 1537 (85.7) | 319 (85.3) | 532 (91.3) | 215 (92.7) |
| Sex, n (%) |  |  |  |  |
| Male | 1143 (63.7) | 250 (66.8) | 393 (67.4) | 146 (62.9) |
| Female | 650 (36.3) | 124 (33.2) | 190 (32.6) | 86 (37.1) |
| Onset age（d）, median (IQR) | 5.07 (5.04, 5.46) | 5.20 (5.03, 12.25) | 50.56 (39.36, 67.69) | 55.14 (42.51, 74.66) |
| Gestational age, n (%) |  |  |  |  |
| 28-32W | 484 (27.0) | 132 (35.3) | 68 (11.6) | 35 (15.0) |
| 32-37W | 553 (30.8) | 79 (21.1) | 69 (11.8) | 21 (9.1) |
| >37W | 756 (42.2) | 163 (43.6) | 446 (76.5) | 176 (75.9) |
| Birth weight, n (%) |  |  |  |  |
| <1500g | 363 (20.2) | 103 (27.5) | 124 (21.3) | 56 (24.1) |
| 1500-2500g | 616 (34.4) | 71 (19.0) | 114 (19.6) | 44 (19.0) |
| 2500-4000g | 762 (42.5) | 193 (51.6) | 324 (55.6) | 123 (53.0) |
| >4000g | 52 (2.9) | 7 (1.9) | 21 (3.6) | 9 (3.9) |
| Mode of delivery, n (%) |  |  |  |  |
| Cesarean delivery | 1444 (80.5) | 287 (76.7) | 164 (28.1) | 74 (31.9) |
| Vaginal birth | 349 (19.5) | 87 (23.3) | 419 (71.9) | 158 (68.1) |
| Feeding, n (%) |  |  |  |  |
| Breastfeeding | 36 (2.0) | 77 (20.6) | 53 (9.1) | 41 (17.7) |
| Formula | 1666 (92.9) | 286 (76.5) | 502 (86.1) | 171 (73.7) |
| Mixture | 91 (5.1) | 11 (2.9) | 28 (4.8) | 20 (8.6) |
| Fever, n (%) |  |  |  |  |
| Yes | 56 (3.1) | 24 (6.4) | 98 (16.8) | 38 (16.4) |
| No | 1737 (96.9) | 350 (93.6) | 485 (83.2) | 194 (83.6) |
| Breathlessness, n (%) |  |  |  |  |
| Yes | 1124 (62.7) | 200 (53.5) | 86 (14.8) | 34 (14.7) |
| No | 669 (37.3) | 174 (46.5) | 497 (85.2) | 198 (85.3) |
| Vomiting, n (%) |  |  |  |  |
| Yes | 122 (6.8) | 27 (7.2) | 53 (9.1) | 16 (6.9) |
| No | 1671 (93.2) | 347 (92.8) | 530 (90.9) | 216 (93.1) |
| Abdominal distension, n (%) |  |  |  |  |
| Yes | 90 (5.0) | 26 (7.0) | 39 (6.7) | 19 (8.2) |
| No | 1703 (95.0) | 348 (93.0) | 544 (93.3) | 213 (91.8) |
| Cyanosis, n (%) |  |  |  |  |
| Yes | 218 (12.2) | 74 (19.8) | 48 (8.2) | 9 (3.9) |
| No | 1575 (87.8) | 300 (80.2) | 535 (91.8) | 223 (96.1) |
| Hepatomegaly, n (%) |  |  |  |  |
| Yes | 1195 (66.6) | 214 (57.2) | 351 (60.2) | 137 (59.1) |
| No | 598 (33.4) | 160 (42.8) | 232 (39.8) | 95 (40.9) |
| Spleomegaly, n (%) |  |  |  |  |
| Yes | 163 (9.1) | 38 (10.2) | 56 (9.6) | 26 (11.2) |
| No | 1630 (90.9) | 336 (89.8) | 527 (90.4) | 206 (88.8) |
| Intubation, n (%) |  |  |  |  |
| Yes | 303 (16.9) | 155 (41.4) | 44 (5.4) | 38 (4.7) |
| No | 1490 (83.1) | 219 (58.6) | 539 (66.1) | 194 (23.8) |
| CHD, n (%) |  |  |  |  |
| Yes | 642 (35.8) | 148 (39.6) | 50 (8.6) | 42 (18.1) |
| PDA | 498 (27.8) | 116 (31.0) | 9 (1.5) | 8 (3.4) |
| VSD | 44 (2.5) | 11 (2.9) | 13 (2.2) | 11 (4.7) |
| ASD | 79 (4.4) | 17 (4.5) | 23 (3.9) | 19 (8.2) |
| Others | 21 (1.2) | 4 (1.1) | 5 (0.9) | 3 (1.3) |
| No | 1151 (64.2) | 226 (60.4) | 533 (91.4) | 190 (81.9) |
| Intracranial haemorrhage, n (%) |  |  |  |  |
| Yes | 113 (6.3) | 23 (6.1) | 28 (4.8) | 11 (4.7) |
| No | 1680 (93.7) | 351 (93.9) | 555 (95.2) | 221 (95.3) |
