# Supplementary material for: Retrospective assessment of the predictors of neonatal and infantile cholestasis with and without liver failure: an experience from Southeast China
Source: PeerJ. 2026 Feb 10;14:e20800. doi: 10.7717/peerj.20800 (PMC12903896; doi:10.7717/peerj.20800)
Supplement: Supplemental Information 3 — CMV: Cytomegalovirus; Ca: Calcium; K: Kalium; Mg: Magnesium; Na: Natrium; P: Phosphorus; ALP: Alkaline phosphatase; ALT: Alanine aminotransferase; AST: Aspartate aminotransferase; DBil: Direct dilirubin; GGT: Gamma-glutamyl transpeptidase; IBil: Indirect bilirubin; TBA: Total bile acid; TBil: Total bilirubin; HDL: High density lipoprotein; LDL: Low density lipoprotein; CK: Creatine kinase; CKMB: Creatine kinase-MB; LDH: Lactate dehydrogenase; MCHC: Mean corpuscular haemoglobin concentration; RDWSD: Red cell distribution width- standard deviation; MCV: Mean corpuscular volume; PLT: Platelet; CRP: C-reactive protein ; Fib: Fibrinogen. [file peerj-14-20800-s003.docx]

| **Characteristics** | **Neonatal group** | | **Infantile group** | |
| --- | --- | --- | --- | --- |
|  | **Development cohort (n=1793)** | **Validation cohort (n=374)** | **Development cohort (n=583)** | **Validation cohort (n=232)** |
| CMV infection, n (%) |  |  |  |  |
| Yes | 86 (4.8) | 44 (11.8) | 207 (35.5) | 89 (38.4) |
| No | 1707 (95.2) | 330 (88.2) | 376 (64.5) | 143 (61.6) |
| Ca, median (IQR) | 2.21 (2.08, 2.35) | 2.30 (2.11, 2.44) | 2.36 (2.22, 2.48) | 2.40 (2.25, 2.54) |
| K, median (IQR) | 4.40 (4.00, 4.80) | 4.40 (4.00, 4.90) | 5.00 (4.50, 5.50) | 4.90 (4.50, 5.50) |
| Mg, median (IQR) | 0.83 (0.75, 0.91) | 0.80 (0.69, 0.89) | 0.93 (0.87, 1.00) | 0.89 (0.80, 0.97) |
| Na, median (IQR) | 138.00 (136.00, 141.00) | 138 .00(135.80, 140.00) | 137.00 (135.00, 138.00) | 137.00 (135.00, 138.50) |
| P, median (IQR) | 1.83 (1.52, 2.12) | 1.77 (1.45, 2.04) | 1.80 (1.53, 2.01) | 1.83 (1.53, 2.01) |
| ALP, median (IQR) | 176.00 (132.50, 243.10) | 207.65 (151.15, 318.05) | 331.20 (247.65, 475.60) | 413.45 (277.15, 542.00) |
| ALT, median (IQR) | 9.40 (5.40, 16.80) | 10.30 (5.99, 20.48) | 49.70 (22.80, 99.60) | 66.02 (29.76, 143.01) |
| AST, median (IQR) | 31.40 (20.60, 51.15) | 29.20 (20.90, 50.33) | 69.60 (39.80, 124.80) | 88.50 (47.30, 192.23) |
| DBil, median (IQR) | 20.40 (19.10, 23.60) | 19.80 (18.10, 28.70) | 27.80 (19.30, 56.30) | 49.60 (20.83, 82.63) |
| GGT, median (IQR) | 135.30 (84.65, 222.40) | 142.95 (81.50, 245.45) | 139.00 (84.90, 254.70) | 141.90 (78.48, 249.52) |
| IBil, median (IQR) | 35.70 (22.70, 84.05) | 42.00 (21.98, 82.23) | 20.60 (10.50, 37.40) | 24.80 (13.75, 42.60) |
| Prealbumin, median (IQR) | 9.16 (7.57, 11.01) | 9.47 (7.53, 11.53) | 11.57 (8.36, 14.50) | 11.35 (6.69, 14.20) |
| TBA, median (IQR) | 10.70 (5.60, 17.40) | 23.72 (12.30, 45.94) | 50.00 (18.20, 83.00) | 92.34 (50.00, 134.50) |
| TBil, median (IQR) | 60.05 (44.95, 110.08) | 69.45 (44.15, 121.95) | 60.90 (36.50, 84.20) | 80.05 (43.00, 129.08) |
| Total protein, median (IQR) | 48.60 (43.80, 53.80) | 47.20 (42.10, 53.33) | 55.10 (50.40, 59.70) | 53.30 (48.63, 58.88) |
| Globulin, median (IQR) | 16.50 (13.60, 20.00) | 15.75 (12.90, 18.60) | 17.30 (14.80, 21.70) | 15.90 (13.50, 19.50) |
| Cholesterol, median (IQR) | 2.16 (1.65, 2.78) | 2.47 (1.96, 3.27) | 3.32 (2.67, 4.05) | 3.75 (2.91, 4.94) |
| HDL, median (IQR) | 0.83 (0.64, 1.05) | 0.63 (0.41, 0.93) | 0.89 (0.64, 1.22) | 0.60 (0.41, 0.93) |
| LDL, median (IQR) | 0.93 (0.59, 1.37) | 1.06 (0.75, 1.53) | 1.62 (1.18, 2.13) | 2.05 (1.40, 2.80) |
| Triglyceride, median (IQR) | 0.35 (0.20, 0.63) | 0.83 (0.54, 1.32) | 1.15 (0.69, 1.74) | 1.40 (0.95, 2.06) |
| CK, median (IQR) | 188.90 (96.00, 412.90) | 151.60 (85.80, 326.65) | 104.80 (69.90, 158.40) | 109.00 (67.88, 169.68) |
| CKMB, median (IQR) | 39.90 (26.00, 77.50) | 39.25 (25.90, 66.80) | 32.10 (23.40, 47.80) | 40.55 (29.13, 58.23) |
| LDH, median (IQR) | 421.00 (318.15, 580.35) | 406.95 (315.40, 574.58) | 323.70 (265.30, 437.16) | 347.90 (282.00, 458.75) |
| Urea, median (IQR) | 3.60 (2.59, 4.88) | 3.35 (2.24, 4.96) | 2.87 (2.07, 3.80) | 3.25 (2.40, 4.30) |
| Creatinine, median (IQR) | 56.20 (41.80, 72.50) | 33.80 (24.68, 49.90) | 28.50 (22.00, 42.10) | 20.20 (15.93, 24.60) |
| Uric acid, median (IQR) | 168.00 (100.00, 294.00) | 144.70 (97.88, 252.58) | 149.00 (112.00, 203.00) | 175.30 (137.18, 227.25) |
| Leucocyte, median (IQR) | 11.98 (9.05, 16.14) | 10.48 (8.24, 13.90) | 10.11 (7.86, 13.23) | 10.37 (8.01, 13.25) |
| Neutrophil, median (IQR) | 10.40 (9.70, 11.20) | 10.40 (9.26, 11.50) | 10.40 (9.70, 11.20) | 9.60 (8.20, 10.93) |
| Lymphocyte, median (IQR) | 4.18 (3.16, 5.39) | 4.09 (3.21, 5.27) | 5.36 (3.83, 7.24) | 5.48 (4.01, 7.44) |
| Eosinophils, median (IQR) | 0.35 (0.17, 0.61) | 0.41 (0.19, 0.69) | 0.28 (0.12, 0.50) | 0.33 (0.18, 0.52) |
| Hematokrit, median (IQR) | 42.40 (35.40, 49.50) | 37.10 (29.95, 45.00) | 30.70 (27.60, 34.00) | 30.55 (27.30, 34.50) |
| Haemoglobin, median (IQR) | 146.00 (121.00, 171.00) | 128.00 (103.00, 155.25) | 103.00 (91.00, 114.00) | 104.00 (93.00, 116.00) |
| Erythrocyte, median (IQR) | 4.29 (3.62, 4.97) | 3.89 (3.20, 4.58) | 3.48 (3.11, 3.85) | 3.47 (3.12, 3.93) |
| MCHC, median (IQR) | 346.00 (332.00, 357.00) | 346.00 (335.75, 358.00) | 336.00 (324.00, 348.00) | 339.00 (329.00, 348.00) |
| RDWSD, median (IQR) | 56.30 (51.10, 62.14) | 55.70 (51.08, 62.00) | 48.80 (44.33, 54.00) | 50.50 (46.33, 55.93) |
| MCV, median (IQR) | 99.10 (93.70, 104.65) | 96.10 (91.18, 101.53) | 88.80 (85.10, 93.10) | 89.00 (85.05, 92.50) |
| PLT, median (IQR) | 263.00 (186.00, 353.00) | 299.50 (206.00, 387.50) | 387.00 (269.00, 492.00) | 362.50 (275.00, 478.25) |
| CRP, median (IQR) | 0.53 (0.50, 2.10) | 1.73 (0.52, 3.70) | 0.86 (0.50, 4.32) | 1.89 (0.54, 4.46) |
| Blood glugose, median (IQR) | 3.75 (2.71, 4.77) | 4.33 (3.37, 5.55) | 4.69 (3.94, 5.66) | 4.99 (4.24, 5.97) |
| Ddimer, median (IQR) | 2.06 (0.96, 4.56) | 3.80 (1.38, 6.75) | 1.08 (0.52, 2.22) | 2.14 (0.87, 4.56) |
| GGT/Albumin, median (IQR) | 4.18 (2.65, 7.04) | 4.43 (2.83, 7.55) | 3.93 (2.22, 7.32) | 4.07 (2.22, 6.99) |
| GGT/ALT, median (IQR) | 14.64 (7.17, 28.16) | 13.97 (6.08, 28.03) | 3.25 (1.44, 6.44) | 2.17 (1.03, 5.35) |
| Triglyceride/HDL, median (IQR) | 0.43 (0.21, 0.88) | 1.29 (0.72, 2.75) | 1.31 (0.64, 2.48) | 2.24 (1.27, 4.66) |
| Uric acid/Creatinine, median (IQR) | 0.03 (0.01, 0.10) | 4.82 (2.88y, 6.66) | 5.05 (3.20, 7.54) | 8.98 (6.65, 12.10) |
| ALP/Albumin, median (IQR) | 0.07 (0.04, 0.14) | 6.52 (4.59, 10.10) | 9.24 (6.72, 12.48) | 11.36 (7.89, 14.71) |
| PLT/Lymphocyte, median (IQR) | 64.86 (44.41, 90.45) | 74.90 (51.18, 96.00) | 68.77 (44.64, 101.39) | 66.79 (44.68, 94.06) |
| Neutrophi/Lymphocyte, median (IQR) | 2.51 (1.94, 3.28) | 2.48 (1.82, 3.34) | 0.51 (0.36, 0.70) | 0.91 (0.57, 1.17) |
| Albumin/Fib, median (IQR) | 18.19 (12.92, 24.31) | 17.32 (12.57, 24.15) | 17.25 (13.53, 23.54) | 16.54 (12.14, 22.00) |
| AST/PLT, median (IQR) | 0.12 (0.07, 0.24) | 0.11 (0.06, 0.21) | 0.18 (0.11, 0.38) | 0.25 (0.13, 0.59) |
| Lymphocyte/leucocyte, median (IQR) | 0.37 (0.25, 0.48) | 0.41 (0.30, 0.52) | 0.56 (0.45, 0.67) | 0.58 (0.45, 0.67) |
