# Supplementary material for: Retrospective assessment of the predictors of neonatal and infantile cholestasis with and without liver failure: an experience from Southeast China
Source: PeerJ. 2026 Feb 10;14:e20800. doi: 10.7717/peerj.20800 (PMC12903896; doi:10.7717/peerj.20800)
Supplement: Supplemental Information 4 — There were 1793 patients in the development cohort, overlapped diagnoses (n = 5965 ) with a mean of 3.33 major health issues (or diagnoses) were noted per patient. There were 374 patients in the validation cohort, overlapped diagnoses (n = 1329 ) with a mean of 3.55 major health issues (or diagnoses) were noted per patient. TORCH: Toxoplasma, Others, Rubella virus, Cytomegalovirus, Herpes virus; PFIC: Progressive familial intrahepatic cholestasis; NTCP: Sodium taurocholate cotransporting polypeptide; NEC: Neonatal necrotizing enterocolitis. [file peerj-14-20800-s004.docx]

| **Classification** | **Specific issues** | **Development cohort** | | **Validation cohort** | |
| --- | --- | --- | --- | --- | --- |
|  |  | **n** | **%** | **n** | **%** |
| **Structural abnormality** | Biliary atresia | 24 | 1.34% | 18 | 4.81% |
|  | Choledochal cyst | 17 | 0.95% | 12 | 3.21% |
|  | Bile duct sludge | 15 | 0.84% | 6 | 1.60% |
|  | Choledocholithiasis | 3 | 0.17% | 2 | 0.53% |
|  | Annular pancreas | 8 | 0.45% | 3 | 0.80% |
|  | Others | 7 | 0.39% | 3 | 0.80% |
| **Infection** | Pneumonia | 1178 | 65.70% | 226 | 60.43% |
|  | Sepsis | 542 | 30.23% | 100 | 26.74% |
|  | TORCH | 114 | 6.36% | 54 | 14.44% |
|  | Enteritis | 326 | 18.18% | 81 | 21.66% |
|  | Cholangitis | 3 | 0.17% | 1 | 0.27% |
|  | Intracranial infection | 73 | 4.07% | 11 | 2.94% |
|  | Peritonitis | 104 | 5.80% | 39 | 10.43% |
|  | Urinary system infection | 33 | 1.84% | 2 | 0.53% |
|  | Others | 182 | 10.15% | 51 | 13.64% |
| **Genetic/metabolic disorder** | Citrin protein deficiency | 35 | 1.95% | 8 | 2.14% |
|  | Chromosomal disease | 41 | 2.29% | 2 | 0.53% |
|  | PFIC | 5 | 0.28% | 1 | 0.27% |
|  | NTCP | 12 | 0.67% | 2 | 0.53% |
|  | Down's syndrome | 9 | 0.50% | 3 | 0.80% |
|  | Noonan syndrome | 8 | 0.45% | 2 | 0.53% |
|  | Alagille syndrome | 4 | 0.22% | 1 | 0.27% |
|  | Tyrosinemia | 3 | 0.17% | 2 | 0.53% |
|  | Galactosemia | 2 | 0.11% | 1 | 0.27% |
|  | Mitochondrial myopathy | 5 | 0.28% | 1 | 0.27% |
|  | Bile Acid Synthesis Defect | 2 | 0.11% | 1 | 0.27% |
|  | Others | 18 | 1.00% | 1 | 0.27% |
| **Endocrine disorder** | Hypothyroidism | 136 | 7.59% | 50 | 13.37% |
|  | Panhypopituitarism | 1 | 0.06% | 1 | 0.27% |
|  | Others | 5 | 0.28% | 3 | 0.80% |
| **Haematology and malignancy** | Solid tumour | 24 | 1.34% | 5 | 1.34% |
|  | Hemophagocytic syndrome | 2 | 0.11% | 2 | 0.53% |
|  | Hemolytic disease | 82 | 4.57% | 34 | 9.09% |
|  | Lymphoma | 0 | 0.00% | 0 | 0.00% |
|  | Leukaemia | 2 | 0.11% | 2 | 0.53% |
|  | Others | 5 | 0.28% | 3 | 0.80% |
| **Cardiovascular disease** | Kawasaki disease | 0 | 0.00% | 0 | 0.00% |
|  | Congenital heart disease | 642 | 35.81% | 148 | 39.57% |
|  | Others | 25 | 1.39% | 4 | 1.07% |
| **Perinatal issues** | Gestational age <32 week | 484 | 26.99% | 132 | 35.29% |
|  | Birth weight <1500 g | 363 | 20.25% | 103 | 27.54% |
|  | Intracranial haemorrhage | 113 | 6.30% | 23 | 6.15% |
|  | NEC | 278 | 15.50% | 71 | 18.98% |
|  | Birth asphyxia | 475 | 26.49% | 9 | 2.41% |
|  | Others | 59 | 3.29% | 19 | 5.08% |
| **Drug related** | Drug-induced liver injury | 408 | 22.75% | 78 | 20.85% |
| **Idiopathic cholestasis** | Idiopathic cholestasis | 88 | 4.91% | 8 | 2.14% |
