# Supplementary material for: Retrospective assessment of the predictors of neonatal and infantile cholestasis with and without liver failure: an experience from Southeast China
Source: PeerJ. 2026 Feb 10;14:e20800. doi: 10.7717/peerj.20800 (PMC12903896; doi:10.7717/peerj.20800)
Supplement: Supplemental Information 5 — There were 583 patients in the development cohort, overlapped diagnoses (n = 1601) with a mean of 2.75 major health issues (or diagnoses) were noted per patient. There were 232 patients in the validation cohort , overlapped diagnoses (n = 599) with a mean of 2.58 major health issues (or diagnoses) were noted per patient. TORCH: Toxoplasma, Others, Rubella virus, Cytomegalovirus, Herpes virus; PFIC: Progressive familial intrahepatic cholestasis; NTCP: Sodium taurocholate cotransporting polypeptide; NEC: Neonatal necrotizing enterocolitis . [file peerj-14-20800-s005.docx]

| **Classification** | **Specific issues** | **Development cohort** | | **Validation cohort** | |
| --- | --- | --- | --- | --- | --- |
|  |  | **n** | **%** | **n** | **%** |
| **Structural abnormality** | Biliary atresia | 89 | 15.27% | 42 | 18.10% |
|  | Choledochal cyst | 38 | 6.52% | 1 | 0.43% |
|  | Bile duct sludge | 5 | 0.86% | 1 | 0.43% |
|  | Choledocholithiasis | 5 | 0.86% | 2 | 0.86% |
|  | Annular pancreas | 2 | 0.34% | 1 | 0.43% |
|  | Others | 14 | 2.40% | 1 | 0.43% |
| **Infection** | Pneumonia | 239 | 40.99% | 78 | 33.62% |
|  | Sepsis | 38 | 6.52% | 32 | 13.79% |
|  | TORCH | 222 | 38.08% | 101 | 43.53% |
|  | Enteritis | 77 | 13.21% | 19 | 8.19% |
|  | Cholangitis | 34 | 5.83% | 13 | 5.60% |
|  | Intracranial infection | 12 | 2.06% | 3 | 1.29% |
|  | Peritonitis | 8 | 1.37% | 3 | 1.29% |
|  | Urinary system infection | 13 | 2.23% | 4 | 1.72% |
|  | Others | 173 | 29.67% | 50 | 21.55% |
| **Genetic/metabolic disorder** | Citrin protein deficiency | 60 | 10.29% | 15 | 6.47% |
|  | Chromosomal disease | 5 | 0.86% | 1 | 0.43% |
|  | PFIC | 4 | 0.69% | 1 | 0.43% |
|  | NTCP | 7 | 1.20% | 1 | 0.43% |
|  | Down's syndrome | 3 | 0.51% | 1 | 0.43% |
|  | Noonan syndrome | 5 | 0.86% | 1 | 0.43% |
|  | Alagille syndrome | 3 | 0.51% | 0 | 0.00% |
|  | Tyrosinemia | 3 | 0.51% | 0 | 0.00% |
|  | Galactosemia | 2 | 0.34% | 0 | 0.00% |
|  | Mitochondrial myopathy | 3 | 0.51% | 2 | 0.86% |
|  | Bile Acid Synthesis Defect | 1 | 0.17% | 0 | 0.00% |
|  | Others | 5 | 0.86% | 2 | 0.86% |
| **Endocrine disorder** | Hypothyroidism | 8 | 1.37% | 14 | 6.03% |
|  | Panhypopituitarism | 2 | 0.34% | 1 | 0.43% |
|  | Others | 2 | 0.34% | 0 | 0.00% |
| **Haematology and malignancy** | Solid tumour | 7 | 1.20% | 2 | 0.86% |
|  | Hemophagocytic syndrome | 4 | 0.69% | 3 | 1.29% |
|  | Hemolytic disease | 5 | 0.86% | 9 | 3.88% |
|  | Lymphoma | 3 | 0.51% | 0 | 0.00% |
|  | Leukaemia | 2 | 0.34% | 0 | 0.00% |
|  | Others | 1 | 0.17% | 1 | 0.43% |
| **Cardiovascular disease** | Kawasaki disease | 4 | 0.69% | 3 | 1.29% |
|  | Congenital heart disease | 50 | 8.58% | 42 | 18.10% |
|  | Others | 2 | 0.34% | 1 | 0.43% |
| **Perinatal issues** | Gestational age <32 week | 68 | 11.66% | 35 | 15.09% |
|  | Birth weight <1500 g | 124 | 21.27% | 56 | 24.14% |
|  | Intracranial haemorrhage | 28 | 4.80% | 11 | 4.74% |
|  | NEC | 24 | 4.12% | 10 | 4.31% |
|  | Birth asphyxia | 146 | 25.04% | 1 | 0.43% |
|  | Others | 3 | 0.51% | 0 | 0.00% |
| **Drug related** | Drug-induced liver injury | 10 | 1.71% | 9 | 3.88% |
| **Idiopathic cholestasis** | Idiopathic cholestasis | 38 | 6.52% | 26 | 11.21% |
